# Supplementary material for: Usability of mental illness simulation involving scenarios with patients with schizophrenia via immersive virtual reality: A mixed methods study
Source: PLoS One. 2020 Sep 16;15(9):e0238437. doi: 10.1371/journal.pone.0238437 (PMC7494071; doi:10.1371/journal.pone.0238437)
Supplement: S3 Data — (PDF) [file pone.0238437.s003.pdf]

Manuals for devices and how to start the VR simulation program

Device: Oculus go

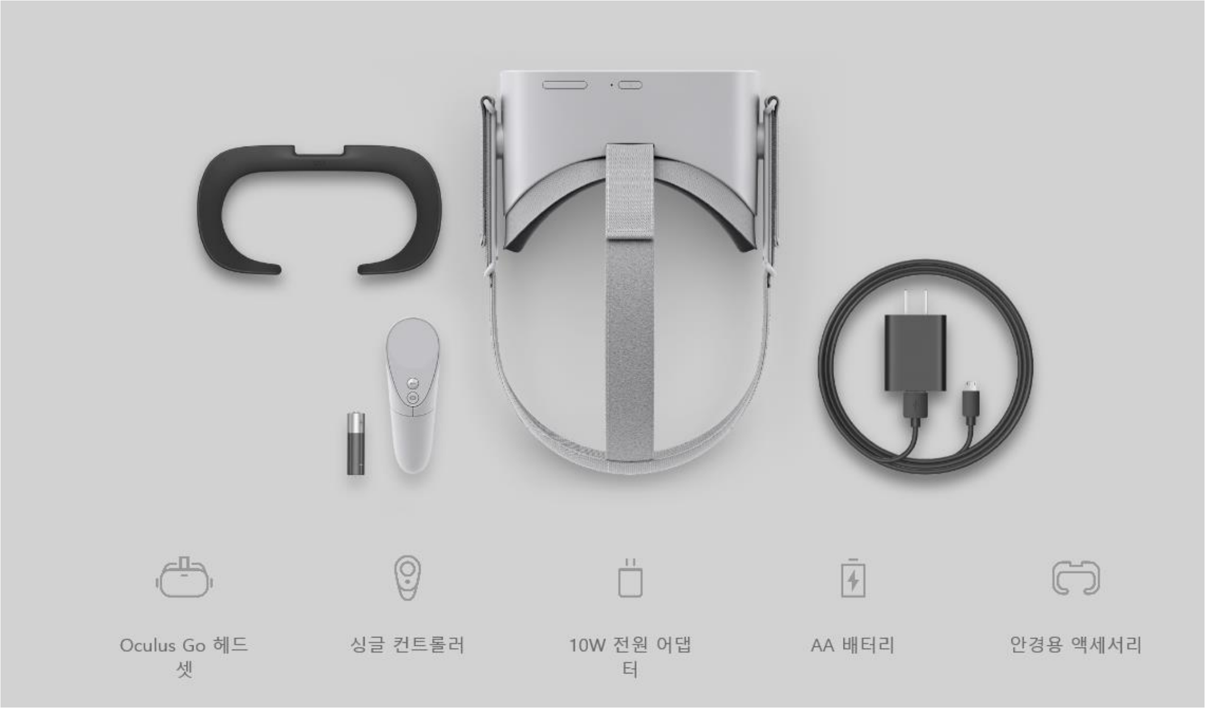

HMD

Controller

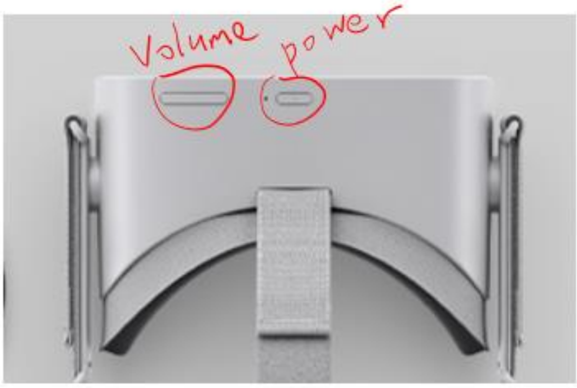

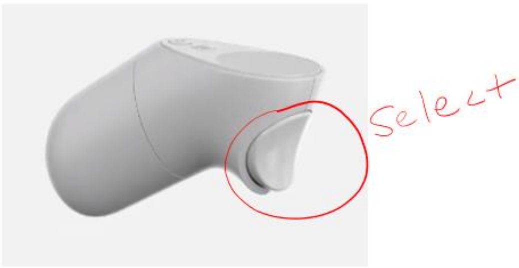

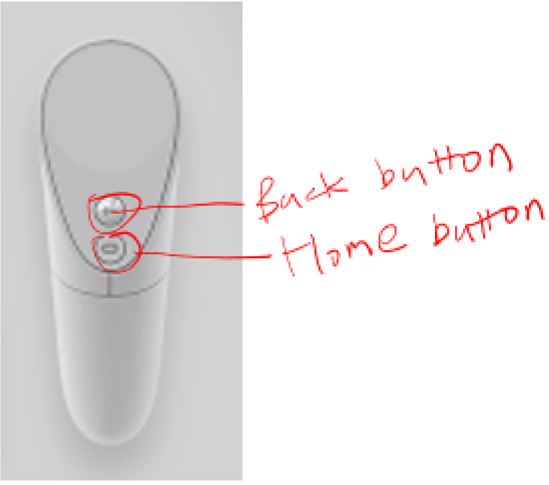

1. Start (in the library): Select “unknown source” -> Select “Scene1” -> Read “description”-> Press the “Back button” -> Play the scene -> Press the “Back button” -> Complete the tasks (quiz)
2. After completing “Scene 1” -> Press the “home button” -> Go to “Library”-> Select “unknown source”-> Select “Scene 2”
3. Repeat for the rest of the scene 3, 4 and 5
