# Supplementary material for: Usability of mental illness simulation involving scenarios with patients with schizophrenia via immersive virtual reality: A mixed methods study
Source: PLoS One. 2020 Sep 16;15(9):e0238437. doi: 10.1371/journal.pone.0238437 (PMC7494071; doi:10.1371/journal.pone.0238437)
Supplement: Attachment — Submitted filename: response to reviewer 0817.docx [file pone.0238437.s005.docx]

| Reviewer #3: This is a useful feasibility study of a VR platform for exposing nursing students to interactions with patients with serious mental illness on an inpatient unit. The study had several strengths. A VR mental health training platform could be useful and easily disseminated; not just for nursing but for other mental health disciplines. It was a significant undertaking to develop the content and programming to create the VR training platform. The number of participants (N=60) was also respectable. The authors also did a good job responding to prior reviewer concerns. A few concerns remain. | Authors response  We appreciate reviewers’ meticulous and constructive comments. We have made revisions accordingly. |
| --- | --- |
| The authors frequently refer to the need for VR skilled actors to simulate the "subtle symptoms" of people with schizophrenia. It is not at all clear what this means. Does this refer to facial expressions that indicate hallucination experiences or paranoia? More could be said about this as it relates to the need for a VR platform. Perhaps the figure showing the manual/remotes (Fig 1) could be replaced with a figure illustrating what the authors mean by this, which would be much more helpful. | We have added a more detailed explanation regarding disease symptoms and related issues in nursing education.   - Introduction   In terms of mental health simulation, the most important learning objectives are for students to identify clinical symptoms and learn how to manage problematic symptoms of mentally ill patients. These symptoms are mostly subtle and demand careful observation of patients’ facial expressions, speech, and behavior. For example, patients with advanced schizophrenia often lack facial expressions, due to symptoms associated with a diminished capacity for emotional expression [26]. This can cause difficulties for inexperienced nurses or nursing students in detecting changes in these patients’ clinical conditions. Thus, good acting that meticulously imitates these symptoms is essential for simulation education. Traditionally, standardized patients (SPs) were hired for mental health nursing simulation to portray patients with mentally illness. However, concerns have remained regarding the considerably high cost and time demands, and frequently changing SPs can exhaust faculties’ resources for repeated training [7-9]. |
| Related to this, the need for a VR training platform is not set up well in the introduction. The authors note in the abstract, discussion and elsewhere that the VR experience was "exciting," and comment on motivation to learn about mental health nursing. Safety of interacting on inpatient units is also noted as a barrier to training, as well as the work involved in repeatedly training simulation actors. Is motivation or interest in mental health nursing a barrier; is there a literature on this? How does VR overcome interest in this by being an "exciting" platform? Are students more competent if trained in VR first? The specific problems with nursing training, and how a VR platform solves these problems, is not clearly explained in the introduction, and this set up should drive a more concise discussion. | We added the following paragraphs to the Introduction and Discussion sections:   - Introduction   Psychiatric nursing has a low profile among undergraduate nursing students and has been reported to be one of the least desirable career choices in the field [4,5]. This lack of interest in mental health nursing has been attributed to anxiety related to working with patients with mental illness and feeling insufficiently prepared to perform this type of clinical work [6]. Thus, a lack of first-hand experience with mental health patients and unfamiliarity with mental disorders may result in stigmatization or fear among nursing students. Simulation has been evidenced as an effective alternative that could enhance clinical experience and maximize learning outcomes in mental health nursing [7-9]. Therefore, using integration strategies to create a rich simulation environment where students find learning interesting could effectively reduce barriers to engaging in mental health nursing practice.   - Discussion   In this study, participants found the VR simulation easy to use, engaging, and exciting. Most participants reported that they believed the content of this study’s VR simulation was highly relevant for their education. Some participants expressed their desire to use this program with longer videos filmed of diverse clinical situations to better prepare themselves for in-person clinical training in psychiatric wards. Previous studies of simulation in mental health nursing have found evidence of improving clinical knowledge and reducing clinical placement anxiety [28]. In terms of training for effective communication, previous methodologies such as high-fidelity simulators have been found to provide relatively fewer beneficial effects due to the characteristics of patients with mental illnesses [29]. Suggestions could be made to provide VR training before students participate in simulations. Knowing that students perceive VR as exciting, the reduced stigma and anxiety that results from the use of VR could produce greater learning outcomes for clinical practice. As an alternative clinical training method, VR technology could effectively increase nursing students’ confidence in their interactions with psychiatric patients. |
| There is very little data in this paper, which is essentially a feasibility study introducing the novel VR training platform. Given this limited data and focus, the manuscript is very long. **A more concise introduction laying out the problems and solutions of VR training as described above, and concise discussion of this and the results would improve the manuscript**. For example, there is a lot of discussion of the readability of the text and details of the platform in the discussion which is not as helpful as a focus on the purpose and acceptability of the VR training platform. | To reflect the reviewer’s comment, we eliminated redundant sentences and a figure, making the Introduction, Results, and Discussion sections more concise. |
| The nursing students enrolled in the study had already completed their clinical placements. This suggests they may have already been exposed to patients with mental disorders, which may have impacted their ratings of the experience. If so, since the VR platform is directed at training new students, this limitation should be mentioned. | To address this issue, the following paragraph has been added as a study limitation:   - Limitation   An additional limitation of this study is that participating nursing students had previously completed their clinical placements. This suggests they may have already been exposed to patients with mental disorders, which may have impacted their ratings of the experience. However, having a well-developed VR simulation with durability and broad application could be a strength. The robust VR simulation program could potentially benefit diverse populations, from inexperienced students to health professionals who need a refresher course. |
